# Sound Event Detection by Pseudo-Labeling in Weakly Labeled Dataset

**DOI:** 10.3390/s21248375

**Published:** 2021-12-15

**Authors:** Chungho Park, Donghyeon Kim, Hanseok Ko

**Affiliations:** Department of Electronics and Electrical Engineering, Korea University Seoul, Seoul 136-713, Korea; pch0606@korea.ac.kr (C.P.); kis6470@korea.ac.kr (D.K.)

**Keywords:** dilated convolution, gated linear unit (GLU), noise label, noise loss, segmentation mask, weakly labeled sound event detection (WSED)

## Abstract

Weakly labeled sound event detection (WSED) is an important task as it can facilitate the data collection efforts before constructing a strongly labeled sound event dataset. Recent high performance in deep learning-based WSED’s exploited using a segmentation mask for detecting the target feature map. However, achieving accurate detection performance was limited in real streaming audio due to the following reasons. First, the convolutional neural networks (CNN) employed in the segmentation mask extraction process do not appropriately highlight the importance of feature as the feature is extracted without pooling operations, and, concurrently, a small size kernel forces the receptive field small, making it difficult to learn various patterns. Second, as feature maps are obtained in an end-to-end fashion, the WSED model would be weak to unknown contents in the wild. These limitations would lead to generating undesired feature maps, such as noise in the unseen environment. This paper addresses these issues by constructing a more efficient model by employing a gated linear unit (GLU) and dilated convolution to improve the problems of de-emphasizing importance and lack of receptive field. In addition, this paper proposes pseudo-label-based learning for classifying target contents and unknown contents by adding ’noise label’ and ’noise loss’ so that unknown contents can be separated as much as possible through the noise label. The experiment is performed by mixing DCASE 2018 task1 acoustic scene data and task2 sound event data. The experimental results show that the proposed SED model achieves the best F1 performance with 59.7% at 0 SNR, 64.5% at 10 SNR, and 65.9% at 20 SNR. These results represent an improvement of 17.7%, 16.9%, and 16.5%, respectively, over the baseline.

## 1. Introduction

The recent development in deep learning field shows advances in the event detection field, such as earthquake detection [1] and sound event detection [2,3]. The sound event detection (SED) field has been a research focus due to as it is used in various real-life applications to recognize a target event and detect the onset and offset times in an audio clip. We encounter a rich variety of sound events in our daily lives, such as “baby cries”, “dog barks”, “phone rings”, “sirens”, and “boiling water”. SED can be applied to varied fields by utilizing such events. For example, SED can not only be used for public security surveillance [4] and, by monitoring animal sounds, the maintenance and preservation of ecosystem environments [5,6] but also for equipment failure monitoring, oil and gas pipeline anomaly detection, and seismic wave acoustic detection [7]. Although video or image-based event detection systems are also applicable to the above situations, systems based on audio-only have several advantages. First, they can be used even in dark environments because they do not require illumination. Second, while sound can penetrate or pass around obstacles, photos and videos are directly disturbed. Third, events, such as fire alarms sounding, are only detectable by sound.

In the deep learning framework, SED was developed by using frame-level annotated data (strongly labeled) which contains the starting and ending point of the target event [8,9]. However, since such deep learning models require frame-by-frame event information, constructing a large amount of data for training proved difficult. To alleviate the above limitation, weakly supervised learning models using weakly labeled data have recently been proposed. Weakly labeled data is learned by multi-label classification using multi-instance learning (MIL) [10] rather than frame-level classification and uses only the presence or absence of an event in the audio clip. The structure of the Weakly labeled data-based SED (WSED) models consists of a classifier and detector that detects the on-off sets of an event in an audio clip. The classifier is a multi-label classifier that has multiple labels for a single audio clip, not a one-hot label as used in general classifiers. One of the WSED studies tried to extend a low-dimensional feature map through transpose convolution neural network to a higher level for frame-by-frame event prediction, such as auto-encoder [11]. However, information and characteristics on the time and frequency axis are lost in the feature extraction process using a convolutional neural network (CNN), and it is difficult to extend from low-dimensional features to high-dimensional ones. To avoid this problem, a method of fitting the frame size to the low-dimensional size without expanding the features was proposed [12]. In addition, good performance was achieved with a structure in which a gated linear Unit (GLU) was applied to the CNN feature extraction process as the learning the activation function [13]. However, the frequency axis dimension reduction method is sensitive to noise and shows performance degradation in noisy environments. To solve this problem, a learning method that maintains the time-frequency dimension was devised [14]. This learning method finds a segmentation mask that separates the target event from the input audio clip. This model calculates the probability that the target event will be present in the audio through global pooling on the last layer without reducing the dimensions of the feature map. However, this model has some limitations.

First, the segmentation mask is extracted through CNN without a pooling operation. In general, CNN consists of a convolution operation using the kernel and a pooling operation that can find important features while reducing dimensions. However, since this model learns while maintaining the dimension of the feature map without a pooling operation, importance is not incorporated into the extracted features. Second, there is a limited receptive field due to the fixed kernel size. The receptive field represents the amount of information the CNN model can contain when training. In general CNN models, the size of the feature map decreases as it passes through the layers using a pooling operation so that the features of various patterns can be learned using only a small kernel. In contrast, this model can only learn a simplified pattern using a 3 x 3 size kernel at all layers without a pooling operation to maintain the same size as the input. Third, in the segmentation mask-based method, as the proposed architecture estimates target segmentation masks from an input feature without any spectral and temporal feature reduction, the noise contents in an input T-F feature would continuously influence the CNN feature extraction process, and they degrade the performance of SED system.

This paper improved the model problem by applying GLU [15], dilated convolution [16], noise label, which is pseudo label, and noise loss. To solve the first problem of being unable to judge the importance of the extracted features, the proposed model adopts GLU. The GLU can determine whether or not the extracted feature is valid information by training the gate through the CNN’s kernel, and is used by replacing the activation function. The gate has a value between 0 and 1 according to a sigmoid function, and the flow of information can be controlled by saving important information and discarding other information through element-wise multiplication with the extracted features. To solve the second problem of over-simplified pattern learning due to the limited receptive area, the proposed model uses dilated convolution. Dilated convolution can widen the receptive field by extending the spacing between weights without increasing the parameters of the model and still maintaining the size of the feature map. Lastly, to reduce the effect of noise, noise labels and noise loss are added. This idea was inspired by the blank label of Connectionist Temporal Classification (CTC) [17], which is used in speech recognizers and applied to SED [18,19]. The CTC improved performance by adding a blank label so that frames corresponding to unnecessary information belong to blank labels to reduce frames corresponding to silence or blank belonging to other classes in the speech recognizer. By doing so, this model adds a noise label, which is pseudo label [20], that can play the same role as the blank label of the CTC, so that background sound and noise do not affect the mask extraction of the target class when the segmentation mask is output. The noise information is extracted by additionally outputting the segmentation mask for the noise label. Furthermore, as the segmentation mask from which the noise component is to be extracted brings unnecessary information to the target event, noise loss is added so that each event mask can contain only important information. An overall summary of the main contribution of this paper is that Constructing a more noise-robust model by adding a noise label that can extract noise segmentation mask and applying a noise loss that allows maximum noise contents extraction. Additionally, to supplement the problems of the previous model, GLU and dilated convolution were applied to improve the performance.

The experiments are performed by using DCASE 2018 dataset [21] task1 and task2 [22]. Task 2 dataset which is for the audio tagging is used for the audio tagging and the frame-level detecting evaluation. For noise data, Task 1 dataset which is the scene classification is augmented with three types of SNR levels (0, 10, and 20 dB). The results show that our proposed pesudo label trick method enhances the robustness of the system in the noisy audio stream over state-of-the-art methods.

Section 2 covers the task approach to WSED and techniques applied in this paper. Section 3 introduces the proposed method and model. Section 4 details the changes to the experimental results resulting from adding the techniques used in this paper one by one. Finally, Section 5 contains the conclusions based on these experimental results.

## 2. Related Work

### 2.1. Multi Instance Learning (MIL)

**Figure 1:**
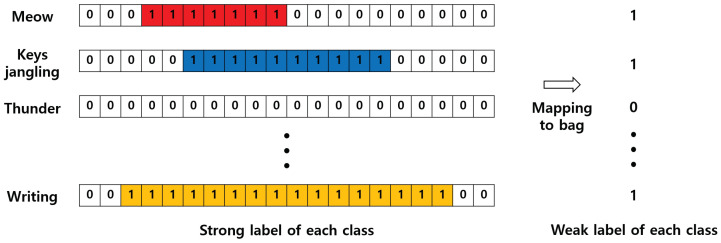
In order to apply WSE to MIL, it is necessary to map the strong label to the weak label through the bag concept. As shown in the picture above, if there is more than one positive value in the strong label, the weak label is mapped to 1; otherwise, it is mapped to 0.

Weakly supervised learning mainly approaches MIL problems [10,23]. It is usually used not only in sound research but also with medical images [24,25] and for semantic segmentation [26,27]. If the concept of MIL is applied to SED, as shown in Figure 1, one audio clip has as many bags as the number of classes, and each bag contains per frame information on whether an event occurs. If one or more frames in the bag have a positive value, then the bag also takes a positive value and is mapped to 1. On the contrary, if there is no event information in any frame in the bag, the bag has a negative value and is mapped to 0. If SED is approached in this way, as a MIL problem through weakly supervised learning rather than through Acoustic Scene Classification (ASC) [28,29] in which audio clips are mapped to one class, the model can be trained through a multiple-instance binary classification that can be mapped to multiple event classes.

### 2.2. Dilated Convolution

**Figure 2:**
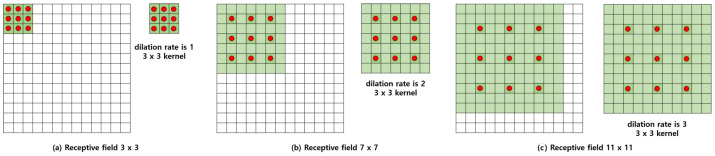
This figure shows how the kernel and receptive field change according to the dilation rate change in dilated convolution. The green area indicates the size of the kernel, and the red dot indicates the learnable parameters. Except for the red dot, the kernel has zero values.

Kernels of different sizes are required to learn the features of varied patterns through CNN. However, when the size of the kernel is increased, the learning speed of the model slows as the number of parameters increases. This problem can be improved by a pooling operation between the CNN layers. When the pooling operation is performed, the size of the feature map is reduced, so the receptive area can be expanded without increasing the size of the kernel. However, as the size of the feature map decreases after the pooling operation, the values excluding specific information disappear, resulting in information loss. This loss of information is fatal for issues, such as image segmentation and image separation, which require the classification of classes by pixel. In this paper, since we also need to learn the segmentation mask of each class in pixel units, we applied dilated convolution [15,30], a method that can learn various patterns while maintaining the information of the feature map. Dilated convolution is already widely used in fields that require classification by pixel [31,32]. As shown in Figure 2, dilated convolution maintains the size of the feature map and expands the distance between the parameters without increasing the number of parameters, meaning that various patterns can be learned as the receptive fields of different size are formed. Red dots indicate parameters that can be learned, and the other areas have a value of 0. The dilation rate refers to the distance between the parameters, and, as is apparent from the figure, the receptive field increases as the dilation rate increases.

### 2.3. Gated Linear Unit (GLU)

**Figure 3:**
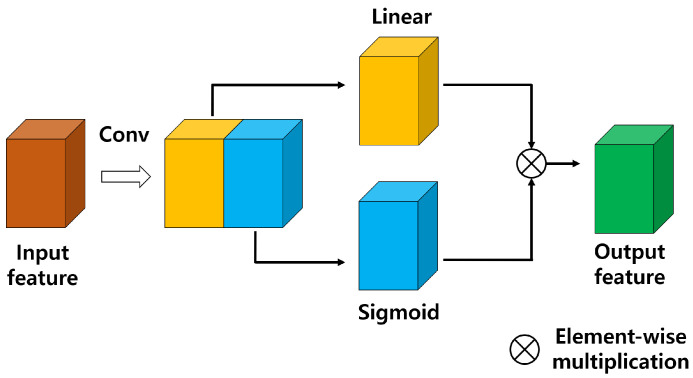
The GLU controls the flow of information by extracting features through the kernel of the convolutional neural network and using one half as information, and the other half as a gate through a sigmoid.

Dilation convolution can be used to learn varied patterns with a small number of parameters without loss of information. However, meaningless features can be extracted because they are learned without the benefit of a pooling operation to determine the importance of pixel information. In this model, information can be controlled according to importance by using a GLU, a learnable activation function [13,16]. A GLU is used as an alternative to the activation function, and, as shown in Figure 3, the front half of each feature extracted through CNN is used as information, and the back half as a gate to determine the importance of the information in the front half. The difference from ReLU [33], which is a commonly used activation function, is that the range of output values is the same, but information flow can be controlled by using a gate. As the gate has a value between 0 and 1 through the sigmoid function, the gate of a pixel with meaningful information has a value close to 1, and the gate of a pixel with meaningless information has a value of 0. The obtained information and the gate control the flow of information through element-wise multiplication so that features can be extracted according to their importance.

## 3. Proposed Method

### 3.1. Extraction Segmentation Mask

**Figure 4:**
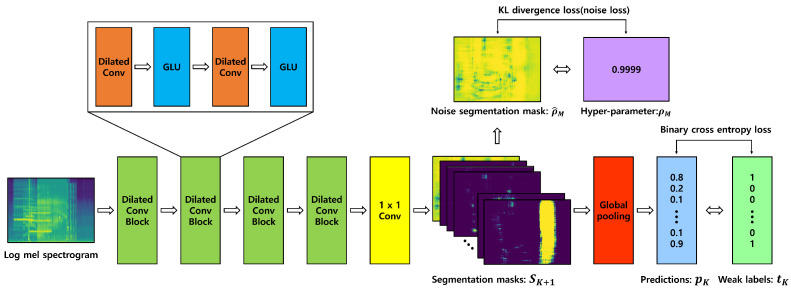
The figure above represents the architecture of the model proposed in this paper. The model receives a log mel spectrogram as input and outputs a segmentation mask through 4 dilated conv blocks and 1 × 1 convolution. The output segmentation mask calculates the probability that an event is included in the audio clip through global pooling. The model is trained using two losses, one of which is binary cross-entropy between predicted probability and weak label, and the other is KL divergence between noise segmentation mask and user hyper-parameter.

To learn the segmentation mask for each event in pixel units from the audio clip, the dilated convolution and GLU described above are used. As shown in Figure 4, the input data is a log mel spectrogram, and features for obtaining a segmentation mask are extracted by repeating a dilated convolution block four times with two dilated convolutions in which the activation function is replaced by a GLU. The proposed model architecture is shown in Table 1. When performing the dilated convolution, the dilation rate was set to 1, 2, 4, and 8, which are the optimal dilation rates obtained by the comparative experiments in paper [34], so the learning is carried out by gradually increasing the distance between the weights. The features obtained through the dilated convolution blocks are reduced dimensions through 1 × 1 convolution using as many kernels as the number of classes, so that S, the segmentation mask for each class, can be extracted. Finally, the extracted S is mapped to a value between 0 and 1 for each pixel through the sigmoid function.

### 3.2. Global Pooling to Predict the Presence or Absence of Target Events

The segmentation mask (S) corresponding to each extracted class predicts whether an event occurs in the audio clip through a global pooling operation. The global pooling used global weighted rank pooling (GWRP) [35], which showed the best performance in the baseline model [14]. GWRP can alleviate the problem of underestimating and overestimating through global max pooling, which transfers only the largest value, and global average pooling, which calculates and transfers the average value [35]. This method assigns different weights according to the values of each pixel of S. By increasing the weight of a pixel with a large value and decreasing the weight of a pixel with a small value, the information on all pixels is optimally reflected in the prediction value that tries to determine whether an event occurrence is an output. It is as shown in Equation (1) below.

(1) $$p_{k}=GWRP\left(S_{k}\right)=\frac{1}{N(r)}\sum_{i=1}^{M}r^{i-1}{\left(S_{k}\right)}_{i}.$$

*S* is the segmentation mask, and *k* is the index of each class. *i* is an index in which each pixel value of *S* is sorted in descending order, and *M* is the number of pixels in time × frequency. *r* is a hyper parameter, set to the value of 0≤r≤1, and $N\left(r\right)=\sum_{i=1}^{M}r^{i-1}$ is a normalization term. In this way, when training the entire model, the value predicted through GWRP calculates the binary cross entropy loss with the weak label. The binary cross-entropy loss is calculated as shown in Equation (3) below.

(2) $$\begin{array}{cc} BCE_{loss}\left(p_{k},t_{k}\right) & =-\sum_{k=1}^{K}t_{k}\log GWRP(S_{k}), \end{array}$$

(3) $$\begin{array}{cc} \; & =-\sum_{k=1}^{K}t_{k}\log p_{k}. \end{array}$$

In Equation (2), *p* denotes the target event probability extracted through GWRP, and *t* denotes the weak label of the input data. *k* is the index of the class.

### 3.3. Noise Label and Noise Loss to Extract Noise Contents

In this paper, a model that could reduce the effect of noise is constructed by adding a pseudo label, which is for classifying unknown contests which is naturally faced in the real stream environment. Furthermore, a loss function is added to extract and separate unknown contents as much as possible through the added pseudo label to build a noise-robust model. Here, the pseudo label is represented by the noise label, and the added loss function is represented by noise loss. Before explaining the noise label and noise loss, let us first think about unknown contents. When recording audio in the real environment, it is inevitably mixed with unknown contents, which is noise, according to the environment. In addition, that noise can be distributed across varied frequency bands depending on the environment. Moreover, it is not known when and what noise will be present. In other words, noise is distributed over the time and frequency axes of the audio clip. Considering these characteristics of noise, noise label and noise loss are applied to this model. The noise label was inspired by the blank label of CTC [17]. The blank label of CTC is a label added to prevent confusion in model training due to the prediction of blank and silent frames that are not included in any class of the speech recognizer model. Using this approach, our method predicts and labels noise that is not included in any target events with a noise label. The noise label maps to 1 according to the characteristics of the noise that can be included anywhere in the aforementioned audio clip. That is, the model is trained by adding a noise label mapped to 1 to the weak labels of the event classes constituting the audio clip. To calculate the noise loss that helps to extract the noise as much as possible, first, a noise segmentation mask corresponding to the noise label is additionally extracted during the segmentation mask extraction process. The noise segmentation mask extracted calculates the KL divergence loss for each pixel so that information about noise can be maximally contained and reflect the characteristics of noise that may be distributed over all time-frequency axes. KL divergence is used since it allows a particular probability distribution to be converged to the desired distribution, and the output value of the noise segmentation mask to be given a value between 0 and 1 through the sigmoid. If the mean square error (MSE) function is used, the model does not train well due to the small gradient, so the learning speed of the model is optimized through KL divergence with a large gradient value. Then, the noise loss Equation (4) is as follows.

(4) $$\begin{array}{cc} N_{loss}\left({\hat{\rho }}_{M},\rho \right) & =\frac{1}{M}\sum_{m=1}^{M}\rho \log\frac{\rho }{{\hat{\rho }}_{m}}+\left(1-\rho \right)\log\frac{1-\rho }{1-{\hat{\rho }}_{m}}. \end{array}$$

In the formula, *M* is a time × frame, which means the total number of pixels, ρ, is a hyperparameter that is designated to converge the probability distribution to the desired value. In this model, ρ is 0.9999. $\hat{\rho }$ is the value of each pixel in the output noise segmentation mask. That is, the noise loss plays the role of converging the output value to close to 1 so that the noise segmentation mask can extract noise information that can be maximally distributed to all pixels. As a result, during the segmentation mask extraction process, the event segmentation mask can be obtained with minimal noise influence since noise is separately extracted. Therefore, the losses used when training the model proposed in this paper are the $BCE_{loss}$ and the $N_{loss}$, also proposed in this paper. To define Loss, it is as shown in Equation (5) below.

(5) $$L_{total}=BCE_{loss}+N_{loss}.$$

## 4. Experiment

### 4.1. Database

The database used in the experiment is the public data used in DCASE 2018 task1 [21] and task2 [22]. Task 1 is a scene classification that classifies the environment to which the audio clip corresponds, and 8640 recordings consisting of 10 classes recorded in 10 cities were used. Task 2 is audio tagging that determines whether there is an event in an audio clip. The data is composed of 41 event classes, which vary in length from less than 1 s to longer than 30 s. To proceed with the SED experiment, data was generated using task 1 data as background sounds and task 2 data as event sounds. Before generating the data, several pre-processing tasks were performed. The task 1 data to be used as the background sound was divided so that no background sound was included in both the training and test sets, and task 2 data longer than 4 s to be used as event data was randomly cut to a length between 2 and 4 s. We use only the manually verified audio clips from Task 2 as sound events because the remaining audio clips are unverified and may contain noisy labels. In addition, since the volume of data differs for each event class, the classes comprised of only a small amount of data were made to have at least 600 data items through time-shifting, and it was ensured that there was no event overlap between the training and test sets. The data is generated by randomly selecting the background sound, which is noise, and also picking an arbitrary number of events to be mixed with the background sound and adding to the background sound. For the noise-dependent experiment, data corresponding to SNRs of 0, 10, and 20 are generated by calculating the SNR between the average amplitude of the randomly selected events and the amplitude of the background sound. The generated audio clips contain at least 3 to 8 events, and the training set includes 8000 samples divided into 4 cross-validation folds. Thus, 2000 model evaluation test sets are made for performance measurement.

### 4.2. Feature Extraction

The sampling rate of the audio clip was 32 Khz, and the spectrogram was extracted using short-time Fourier transform(STFT) with a window size of 2048 and a hop size of 1024 since this configuration is known to have a good resolution in the time-frequency domain [36]. As for the generated spectrogram, the mel spectrogram was extracted using 64 mel filter banks, and the log-mel spectrogram was obtained through log operation and used as input. Log-mel spectrograms are widely used in acoustic studies [32,37].

### 4.3. Evaluation and Metric

As evaluation indicators, F1 score [38], Area Under the Curve (AUC) [39], and mean Average Precision (mAP) [40] were used to verify the performance of Audio Tagging (AT) and SED. The F1 score is calculated based on the precision representing the accuracy from the system perspective and the recall representing the accuracy from the data perspective. The F1 score derives a value close to 0 when either the precision or recall has a low value, and a value close to 1 when both have high values.

The AUC is the area under the Receiver Operating Characteristic (ROC) curve, which plots the true positive rate and the false positive rate, and is expressed as a single value. When using the AUC, there is no need to manually designate a threshold, and, when the system outputs a random value, such as when the system is not trained, it has a value of 0.5.

As with AUC, AP refers to the area under the graph for precision and recall. As with the F1 score, the higher the precision and reproducibility, the closer to 1, and, in the case of multiple event detection instead of single-event detection, the performance is evaluated according to the AP average (mAP) of each class.

### 4.4. Post Processing for Performance Evaluation

Post-processing for performance is the same as in the baseline paper extracting the segmentation mask [14]. The performance for AT is measured by determining that there is an event if the probability that each event derived through GWRP is in the audio clip is 0.2 or more, and, if it is less than 0.2, there is no event. It is expressed as (7) in the formula.

(6) $$e_{k}=\left\{\begin{array}{cc} 1 & p_{k}>0.2\\ 0 & otherwise \end{array}\right..$$

$p_{k}$ is the probability for the event class calculated by Equation (1), and *k* is the index indicating event class. The SED predicts the onset of an event when the average sum of information on the frequency axis included in time *t* is 0.1 or more in the segmentation mask and the event offset when it falls below. In addition, the SED result was measured by considering only the case of the class determined to have an event in the AT result. The equations for determining whether an event occurs for time *t* are as in (8) and (9), respectively.

(7) $$q_{k}\left(t\right)=\frac{1}{N}\sum_{i=1}^{N}S_{k}\left(t,f_{i}\right),$$

(8) $$d_{k_{t}}=\left\{\begin{array}{cc} 1 & q_{k}>0.1\\ 0 & otherwise \end{array}\right..$$

*N* is the size of the frequency axis, *t* is the time, *S* is the segmentation mask, and *k* is the number of classes. That is, $q_{k}\left(t\right)$ is the probability that an event occurs at time *t* for the $k_{th}$ class, and $d_{k_{t}}$ indicates whether an event occurs at time *t* of the $k_{th}$ class.

### 4.5. Model

This section describes in detail the architecture of the model proposed in this paper and the baseline model. As shown in Table 1, the input data is log mel spectrogram, and the proposed model used dilated convolution while the baseline used convolution. The kernel used is a constant size of 3 × 3 in all layers of both models, and the number of kernels is 64, 128, 256, and 256. The dilation rates of the proposed model are set to 1, 2, 4, and 8. The activation function uses ReLU in the baseline and GLU in the proposed model. The convolution operation is repeated twice in each kernel, that is, a total of 8 times. The features extracted through the convolution operation reduce the number of channels by the number of event classes through 1 × 1 convolution. In the proposed model, the segmentation mask corresponding to the added noise label is additionally extracted, making one more output channel than the number of classes. The segmentation mask extracted through 1 × 1 convolution passes through the sigmoid and then calculates the probability of each event in the audio clip through GWRP. When training the model, the batch size is 8, the learning rate is 0.001, and the Adam optimizer [41] is used. In addition, batch normalization [42] is applied to all convolution layers except the 1 × 1 convolution to stabilize the model and improve learning speed. The GWRP’s hyperparameter *r* is set to 0.9998.

### 4.6. Ablation Analysis

In this part, we analyze how the performance changes when dilated convolution, GLU, noise label, and noise loss, the techniques used in this paper, are added to the baseline model. The experimental results analyzed are the F1, AUC, and mAP scores of Audio Tagging (AT) and Sound Event Detection (SED) at differing SNRs. In addition, it shows the F1 score for each event class when the SNR is 0 and the output segmentation mask.

#### 4.6.1. Effect of Applying GLU

The reason for applying the GLU is that the CNN, which extracts the segmentation mask from the baseline model, proceeds without pooling so that the importance of the feature is not considered. The experiment was carried out by applying GLU to all convolution layers except the 1 × 1 convolution. Looking at the experimental results in Table 2 and Table 3, AT performance was slightly higher than baseline across all performance indicators and all SNRs, while SED performance differed only slightly at SNRs of 0 or 10, but, at 20, the performance indicators excluding AUC were higher than baseline.

#### 4.6.2. Effect of Applying Dilated Convolution

The reason for using dilated convolution is to expand the receptive field without losing information about the input data while retaining the number of parameters in baseline. The convolution layers of the baseline model except the 1 × 1 convolution were replaced by dilated convolution. Table 2 and Table 3 show that at all SNRs AT and SED show significantly higher performance than baseline. This indicates that for SED the receptive field plays a very important role in the segmentation mask extraction-based method.

#### 4.6.3. Effects of Simultaneous Application of GLU and Dilated Convolution (DCGLU)

This experiment applies Dilated convolution and GLU together. As in the above experiments, the convolution layer of the baseline model was replaced with a dilated convolution layer, and the activation function was changed to GLU. For the proposed model, as shown in Table 2 and Table 3, all indicators except for AUC at SNR 10 show better performance than when only dilated convolution is applied. Since GLU, which is a learnable activation function, is learned through CNN in the same way as the general features, the degree of the effect differs according to the size of the receptive field.

#### 4.6.4. Change When Noise Label Is Added

**Figure 5:**
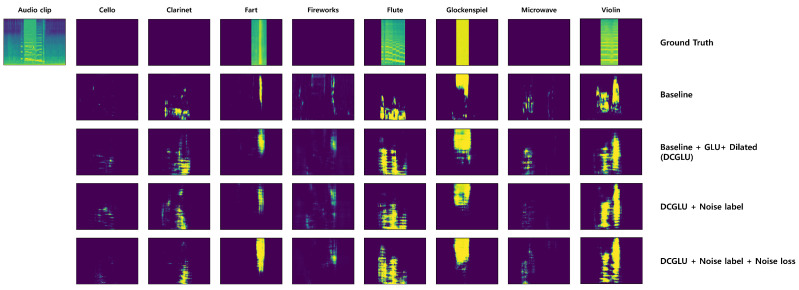
Changes in the output of the segmentation mask as the techniques proposed in this model are applied to the baseline. Ground truth is the log mel spectrogram extracted from each event.

This experiment added a noise label to the DCGLU model. It was conducted to determine the effect of extracting the noise segmentation mask by adding only the noise label without noise loss. Looking at the difference from the DCGLU model in the experimental results presented in Table 2 and Table 3 indicates that the results do not differ significantly with SNRs. There is almost no difference in the average F1 score performance of the entire class in Table 4 and Table 5. As for events with a slight difference, the DCGLU model showed somewhat higher AT and SED performance in events distributed in low frequency bands, such as bass drum, chime, and double base. However, with the model to which the noise label was added, AT and SED performance were better for events occupying a rather wide frequency band, such as cough and shatter, or a high frequency band, such as snare drum. In some cases, in Figure 5, events not included in the audio clip are extracted from the segmentation mask output when the noise label is added, but compared to other models the microwave has the cleanest output. That is, although, when the noise label is added, the effect is insignificant, event and noise are nevertheless separated. Performance is different for each event class because noise is heavily distributed in the low frequency band, and, so, for events occupying a low frequency band, the separation from noise causes more performance degradation.

#### 4.6.5. Validity of Noise Label and Noise Loss

**Figure 6:**
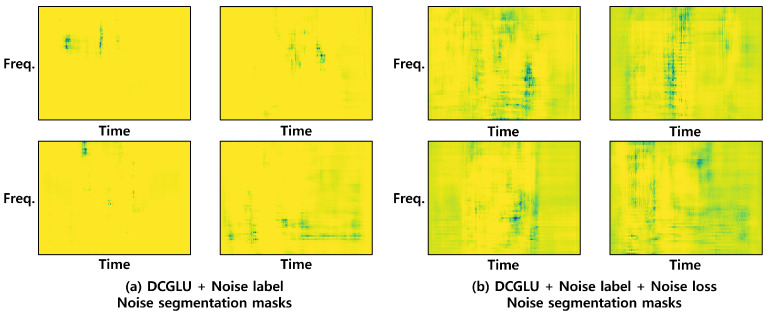
The noise segmentation mask of the model to which only the noise label is applied, and the model to which both the noise label and noise loss are applied. The yellow areas are judged to have noise and represent values closer to 1, and the darker areas values closer to 0.

This experiment adds noise label and noise loss to the DCGLU model. Noise loss is added to extract as much noise information as possible into the noise segmentation mask. Figure 6 shows the extracted noise. Figure 6a is the noise information obtained by adding only a noise label to the DCGLU model, and Figure 6b is the noise information obtained by applying both noise label and noise loss. In Figure 6, yellow represents a value closer to 1, and the darker color a value closer to 0. Comparing the two figures, it seems that the model to which only the noise label was added extracts information about noise from all pixels. On the other hand, for the model to which both the noise label and noise loss are applied, less noise is extracted than the model to which only the noise label is added. However, looking at Figure 5, you can see that the model using noise label and noise loss together extracts each event segmentation mask better than other models, and produces the clearest output for Cello, Clarinet, and Fireworks events, which are absent from the audio clip. Therefore, the phenomenon shown in Figure 6 seems to reflect the event and the noise being rather effectively separated and maximally extracting noise through noise loss.

## 5. Discussion

This is the experimental result of the model that added both noise label and noise loss to the DCGLU proposed in this paper and the weakly labeled data-based model, including the baseline proposed previously. As with the previously proposed models, we experimented with FrameCNN, which is an autoencoder model [11], a WLDCNN model in which all layers are convolutional [12], and an attention model that combines CNN and biRNN, and applies GLU [13]. As Table 2 shows, the attention model demonstrated the highest F1 performance, that is, 0.648 when SNR was 0, 0.695 when it was 10, and 0.698 when it was 20. However, its F1 performance of SED, as shown in Table 3, was lower than for other models, including the baseline. On the other hand, the proposed model, showed better AUC and mAP AT performance, than the Attention model, as Table 2 shows. Furthermore, the proposed model demonstrated the best SED performance across all indicators and all SNRs. AT performance is high for the attention model because global max pooling is used in its last layer to determine the presence of audio events. However, its SED performance is lower than that of the other models because it does not perform backpropagation well due to the effect of global max pooling, which delivers only the largest value. In the case of SED, the proposed model shows the F1 best performance, 0.543 at 0 SNR, 0.595 at 10 SNR, and 0.610 at 20 SNR. This represents increases compared to the baseline of 0.177, 0.169, or 0.165, respectively, with the largest difference at 0 SNR where the most noise was mixed. As for performance by class, in terms of AT, the proposed model outperformed the attention model in 12 event classes, including acoustic guitar and double base, out of a total of 41. However, in the case of SED, the proposed model performed the best across all event classes, as is shown in Table 4 and Table 5. Therefore, considering the above experimental results, it can be seen that other proposed papers are vulnerable to the noise environment and it can be proved that the proposed noise label and noise loss work effectively to distinguish between event contents and noise. Although the performance has been improved, it needs improvement because the performance is not yet usable in a real system. As a future research direction, we plan to apply it to a speaker separation model that extracts a mask, such as a segmentation mask.

## 6. Conclusions

In this paper, a more efficient model was constructed by applying dilated convolution and GLU to improve the lack of receptive field and non-interference of feature importance, which was the problem with the SED models extracting segmentation masks from time-frequency domains. Moreover, a noise-robust model was developed by proposing a noise label that could separate the noise contents in the segmentation mask extraction process and a noise loss that could extract the noise contents to the maximum to solve the performance degradation problem due to noise inevitably mixed with the input data. In the experiment, we proved the performance of the proposed model by showing how the model changed and improved through the performance when the techniques used were applied step by step and the segmentation mask output.

## Figures and Tables

**Table 1:** Comparison of proposed model and baseline model.

Proposed Model			Baseline Model		
**Layers, Activation Function** {**Kernel Size, Dilation Rate, Repeat**}	**Number** **of Kernel**	**Output Size** {**Channel** × **Time** × **Frequency**}	**Layers, Activation Function** {**Kernel Size, Repeat**}	**Number** **of Kernel**	**Output Size** {**Channel** × **Time** × **Frequency**}
Input log mel spectrogram	-	1 × 431 × 64	Input log mel spectrogram	-	1 × 431 × 64
Dilated CNN, GLU {3 × 3, 1, 2}	64	32 × 431 × 64	CNN, ReLU {3 × 3, 2}	64	64 × 431 × 64
Dilated CNN, GLU {3 × 3, 2, 2}	128	64 × 431 × 64	CNN, ReLU {3 × 3, 2}	128	128 × 431 × 64
Dilated CNN, GLU {3 × 3, 4, 2}	256	128 × 431 × 64	CNN, ReLU {3 × 3, 2}	256	256 × 431 × 64
Dilated CNN, GLU {3 × 3, 8, 2}	256	128 × 431 × 64	CNN, ReLU {3 × 3, 2}	256	256 × 431 × 64
CNN, Sigmoid {1 × 1, -, -}	42	42 × 431 × 64	CNN, Sigmoid {1 × 1, -}	41	41 × 431 × 64
Global Weighted Rank Pooling	-	42	Global Weighted Rank Pooling	-	41

**Table 2:** Audio tagging performance according to SNR.

	0 dB			10 dB			20 dB		
**Models**	**F1**	**AUC**	**mAP**	**F1**	**AUC**	**mAP**	**F1**	**AUC**	**mAP**
FrameCNN [11]	0.301	0.675	0.319	0.318	0.696	0.352	0.320	0.708	0.367
WLDCNN [12]	0.289	0.599	0.268	0.312	0.621	0.309	0.300	0.617	0.295
Attention [13]	**0.648**	0.854	0.696	**0.695**	0.874	0.738	**0.698**	0.875	0.743
Baseline [14]	0.423	0.844	0.492	0.463	0.874	0.568	0.471	0.881	0.591
Baseline + GLU	0.427	0.846	0.499	0.465	0.874	0.571	0.482	0.886	0.605
Baseline + Dilated	0.559	0.902	0.687	0.616	0.928	0.758	0.616	0.931	0.764
Baseline + GLU + Dilated (DCGLU)	0.565	0.900	0.687	0.613	0.926	0.752	0.627	0.933	0.776
DCGLU + Noise label	0.565	0.900	0.687	0.614	0.925	0.754	0.628	0.933	0.776
DCGLU + Noise label + Noise loss	0.597	**0.910**	**0.721**	0.645	**0.933**	**0.785**	0.659	**0.940**	**0.802**

**Table 3:** Sound event detection performance according to SNR.

	0 dB			10 dB			20 dB		
**Models**	**F1**	**AUC**	**mAP**	**F1**	**AUC**	**mAP**	**F1**	**AUC**	**mAP**
FrameCNN [11]	0.143	0.621	0.064	0.155	0.638	0.069	0.158	0.648	0.071
WLDCNN [12]	0.105	0.576	0.126	0.146	0.598	0.155	0.121	0.589	0.135
Attention [13]	0.117	0.781	0.218	0.129	0.799	0.235	0.137	0.802	0.242
Baseline [14]	0.366	0.715	0.284	0.426	0.749	0.344	0.445	0.760	0.374
Baseline + GLU	0.366	0.713	0.286	0.425	0.745	0.351	0.452	0.758	0.383
Baseline + Dilated	0.514	0.823	0.473	0.560	0.849	0.526	0.575	0.857	0.545
Baseline + GLU + Dilated (DCGLU)	0.517	0.819	0.471	0.568	0.851	0.533	0.588	0.861	0.558
DCGLU + Noise label	0.516	0.819	0.471	0.570	0.851	0.536	0.587	0.861	0.559
DCGLU + Noise label + Noise loss	**0.543**	**0.832**	**0.502**	**0.595**	**0.861**	**0.564**	**0.610**	**0.870**	**0.583**

**Table 4:** F1 score of audio tagging for each class at SNR 0.

Models	Acous. Guitar	Applause	Bark	Base Drum	Burping	Bus	Cello	Chime	Clarinet	Keyboard	Cough	Cowbell	Double Bass	Drawer	Elec. Piano	Fart	Finger Snap	Fire Works	Flute	Glock.	Gong
FrameCNN [11]	0.275	0.577	0.249	0.288	0.225	0.499	0.345	0.296	0.416	0.217	0.202	0.179	0.218	0.195	0.368	0.233	0.206	0.188	0.423	0.288	0.270
WLDCNN [12]	0.194	0.855	0.195	0.190	0.191	0.589	0.230	0.190	0.468	0.192	0.196	0.190	0.186	0.193	0.252	0.196	0.199	0.196	0.449	0.349	0.194
Attention [13]	0.307	0.866	0.860	0.529	0.787	0.669	0.526	0.693	0.750	0.640	0.783	0.841	0.353	0.337	0.536	0.599	0.711	0.430	0.749	0.534	0.442
Baseline [14]	0.372	0.569	0.518	0.269	0.384	0.441	0.389	0.512	0.446	0.480	0.402	0.345	0.273	0.262	0.422	0.377	0.379	0.273	0.440	0.439	0.348
Baseline + GLU	0.382	0.557	0.514	0.266	0.406	0.432	0.399	0.522	0.453	0.486	0.406	0.346	0.264	0.252	0.420	0.375	0.382	0.270	0.449	0.438	0.355
Baseline + Dilated	0.437	0.763	0.706	0.397	0.558	0.542	0.484	0.604	0.574	0.572	0.555	0.565	0.367	0.314	0.506	0.534	0.494	0.343	0.572	0.567	0.406
Baseline + GLU + Dilated (DCGLU)	0.429	0.746	0.734	0.399	0.560	0.517	0.486	0.646	0.609	0.574	0.566	0.589	0.368	0.312	0.510	0.533	0.505	0.336	0.602	0.582	0.405
DCGLU + Noise label	0.425	0.752	0.737	0.375	0.566	0.513	0.488	0.632	0.599	0.577	0.581	0.602	0.349	0.320	0.492	0.526	0.497	0.328	0.602	0.582	0.404
DCGLU + Noise label + Noise loss	0.466	0.783	0.763	0.431	0.607	0.556	0.494	0.688	0.629	0.586	0.605	0.635	0.380	0.331	0.528	0.599	0.523	0.368	0.627	0.603	0.461
Models	Gunshot	Harmonica	Hihat	Keys	Knock	Laughter	Meow	Microwave	Oboe	Sexophone	Scissors	Shatter	Snare drum	Squeak	Tambourine	Tearing	Telephone	Trumpet	Violin	Writing	Avg.
FrameCNN [11]	0.220	0.559	0.279	0.302	0.175	0.208	0.223	0.285	0.489	0.548	0.231	0.208	0.383	0.202	0.339	0.226	0.312	0.381	0.467	0.213	0.301
WLDCNN [12]	0.245	0.362	0.472	0.197	0.198	0.191	0.200	0.202	0.482	0.702	0.191	0.237	0.296	0.189	0.192	0.197	0.233	0.486	0.495	0.193	0.289
Attention [13]	0.572	0.879	0.924	0.851	0.546	0.651	0.586	0.652	0.678	0.782	0.603	0.597	0.848	0.500	0.753	0.526	0.548	0.845	0.787	0.505	**0.648**
Baseline [14]	0.398	0.532	0.515	0.506	0.327	0.424	0.463	0.360	0.487	0.511	0.404	0.429	0.497	0.362	0.649	0.444	0.388	0.502	0.438	0.378	0.423
Baseline + GLU	0.401	0.555	0.515	0.502	0.320	0.417	0.474	0.371	0.494	0.521	0.409	0.410	0.517	0.374	0.689	0.450	0.387	0.525	0.448	0.373	0.427
Baseline + Dilated	0.514	0.763	0.764	0.731	0.385	0.537	0.611	0.493	0.674	0.636	0.500	0.599	0.655	0.497	0.867	0.559	0.510	0.720	0.638	0.446	0.559
Baseline + GLU + Dilated (DCGLU)	0.505	0.742	0.721	0.740	0.389	0.559	0.646	0.478	0.695	0.660	0.505	0.587	0.639	0.492	0.889	0.575	0.528	0.729	0.643	0.449	0.565
DCGLU + Noise label	0.504	0.746	0.734	0.730	0.382	0.554	0.652	0.484	0.700	0.664	0.495	0.606	0.652	0.509	0.892	0.577	0.525	0.736	0.627	0.445	0.565
DCGLU + Noise label + Noise loss	0.546	0.790	0.765	0.752	0.407	0.588	0.675	0.528	0.730	0.690	0.524	0.642	0.695	0.544	0.892	0.610	0.573	0.746	0.657	0.477	0.597

**Table 5:** F1 score of sound event detection for each class at SNR 0.

Models	Acous. Guitar	Applause	Bark	Base Drum	Burping	Bus	Cello	Chime	Clarinet	Keyboard	Cough	Cowbell	Double Bass	Drawer	Elec. Piano	Fart	Finger Snap	Fire Works	Flute	Glock.	Gong
FrameCNN [11]	0.136	0.343	0.111	0.046	0.071	0.262	0.206	0.157	0.227	0.071	0.064	0.049	0.072	0.061	0.191	0.077	0.055	0.061	0.228	0.132	0.142
WLDCNN [12]	0.000	0.544	0.000	0.000	0.000	0.417	0.113	0.000	0.358	0.000	0.000	0.000	0.002	0.000	0.075	0.060	0.000	0.000	0.283	0.114	0.000
Attention [13]	0.069	0.233	0.076	0.065	0.154	0.189	0.170	0.163	0.231	0.036	0.051	0.070	0.026	0.008	0.127	0.077	0.057	0.023	0.217	0.072	0.110
Baseline [14]	0.290	0.607	0.403	0.149	0.341	0.388	0.346	0.562	0.401	0.457	0.262	0.319	0.062	0.048	0.381	0.305	0.278	0.133	0.401	0.372	0.332
Baseline + GLU	0.296	0.596	0.392	0.151	0.337	0.365	0.347	0.545	0.415	0.453	0.265	0.315	0.054	0.046	0.352	0.305	0.272	0.125	0.423	0.358	0.317
Baseline + Dilated	0.418	0.740	0.637	0.265	0.493	0.520	0.463	0.629	0.589	0.552	0.476	0.507	0.266	0.186	0.505	0.507	0.462	0.241	0.539	0.462	0.388
Baseline + GLU + Dilated (DCGLU)	0.396	0.725	0.657	0.264	0.501	0.471	0.443	0.652	0.596	0.558	0.495	0.535	0.251	0.162	0.498	0.506	0.472	0.216	0.571	0.465	0.375
DCGLU + Noise label	0.388	0.731	0.662	0.245	0.500	0.469	0.448	0.639	0.602	0.554	0.500	0.515	0.232	0.164	0.472	0.499	0.469	0.223	0.575	0.469	0.384
DCGLU + Noise label + Noise loss	0.432	0.753	0.672	0.310	0.538	0.520	0.474	0.672	0.621	0.566	0.532	0.553	0.281	0.177	0.505	0.545	0.505	0.250	0.590	0.477	0.425
Models	Gunshot	Harmonica	Hihat	Keys	Knock	Laughter	Meow	Microwave	Oboe	Sexophone	Scissors	Shatter	Snare drum	Squeak	Tambourine	Tearing	Telephone	Trumpet	Violin	Writing	Avg.
FrameCNN [11]	0.080	0.311	0.129	0.139	0.048	0.081	0.082	0.169	0.310	0.286	0.087	0.076	0.223	0.074	0.208	0.077	0.157	0.214	0.256	0.079	0.143
WLDCNN [12]	0.031	0.264	0.119	0.000	0.000	0.00	0.024	0.100	0.287	0.618	0.003	0.039	0.215	0.000	0.000	0.000	0.000	0.253	0.382	0.000	0.105
Attention [13]	0.056	0.309	0.110	0.098	0.035	0.014	0.054	0.166	0.276	0.294	0.033	0.032	0.149	0.061	0.114	0.031	0.139	0.268	0.269	0.040	0.117
Baseline [14]	0.237	0.593	0.441	0.448	0.159	0.295	0.313	0.341	0.585	0.503	0.349	0.310	0.574	0.287	0.624	0.353	0.363	0.568	0.469	0.346	0.366
Baseline + GLU	0.248	0.622	0.433	0.460	0.153	0.291	0.309	0.319	0.605	0.529	0.350	0.304	0.600	0.298	0.638	0.360	0.361	0.579	0.475	0.360	0.366
Baseline + Dilated	0.431	0.753	0.635	0.682	0.263	0.487	0.517	0.489	0.669	0.627	0.482	0.548	0.671	0.479	0.713	0.515	0.500	0.695	0.637	0.438	0.514
Baseline + GLU + Dilated (DCGLU)	0.424	0.743	0.612	0.695	0.261	0.509	0.568	0.480	0.693	0.646	0.503	0.531	0.677	0.500	0.724	0.520	0.501	0.716	0.641	0.427	0.517
DCGLU + Noise label	0.425	0.752	0.610	0.700	0.254	0.500	0.554	0.485	0.701	0.659	0.506	0.546	0.685	0.492	0.722	0.522	0.515	0.717	0.639	0.435	0.516
DCGLU + Noise label + Noise loss	0.457	0.762	0.644	0.709	0.285	0.534	0.595	0.514	0.706	0.675	0.511	0.562	0.703	0.526	0.725	0.550	0.539	0.728	0.652	0.468	**0.543**

## Data Availability

[dataset] Heittola, Toni; Mesaros, Annamaria; Virtanen, Tuomas. 2018. TUT Urban Acoustic Scenes 2018, Development dataset; Zenodo; Version 1.0; 10.5281/zenodo.1228142, [dataset] Eduardo Fonseca; Xavier Favory; Jordi Pons; Frederic Font; Manoj Plakal; Daniel P. W. Ellis; Xavier Serra. 2019. FSDKaggle2018; Zenodo; Version 1.0; 10.5281/zenodo.2552860.
